# GENDER DIFFERENCES IN THE ASSOCIATION BETWEEN MODIFIABLE RISK FACTORS AND FINANCIAL HARDSHIP

**DOI:** 10.1093/geroni/igz038.3148

**Published:** 2019-11-08

**Authors:** Gillian Marshall, William Bryson, Ola Rostant, Sarah Canham

**Affiliations:** 1 University of Washington, Tacoma, Washington, United States; 2 Oregon Health and Sciences University, Portland, Oregon, United States; 3 University of Michigan - Institute for Social Research, Ann Arbor, Michigan, United States; 4 University of Utah, Salt Lake City, Utah, United States

## Abstract

Objective: To identify associations between modifiable risk factors (cigarette smoking, alcohol consumption, and obesity) and financial hardship (difficulty paying bills, food insecurity and medication need) among middle-aged and older Americans in a nationally representative sample. Methods: This was a cross-sectional study of 8,212 persons age 50 years and older who completed the core 2010 Health and Retirement Study survey and the psychosocial questionnaire. We ran separate multinomial logistic regressions to assess the association of three modifiable risk factors and three different financial hardship indicators. Results: Adjusting for all covariates, compared to men of normal weight, men who were obese had a 1.4 greater odds of difficulty paying their bills (95% CI: 1.08-1.76); former smokers had a 1.8 greater odds of being food insecure (95% CI: 1.05-2.95); current smokers were twice as likely to be food insecure (95% CI: 1.21-3.73); Compared to women who never smoked, current smokers had a 1.5 greater odds of having difficulty paying their bills (95% CI: 1.11-2.02); current smokers had a 1.8 greater odds of being food insecure (95% CI: 1.13-2.91); and women who were obese had a 1.5 greater odds of reducing medication due to cost (95% CI: 1.11, 2.02). Conclusion: Our findings contribute to the literature on health behaviors and financial hardship by highlighting the cyclical nature between different indicators of socioeconomic status, modifiable risk factors, and poor health outcomes among middle-aged and older adults. Furthermore, findings highlight how modifiable risk factors may culminate in financial hardship in later life.

